# The Impact of Augmented Reality-Based Aromatherapy Education on Symptoms in Older Adults with Early-Stage Dementia

**DOI:** 10.3390/healthcare14111482

**Published:** 2026-05-27

**Authors:** Vivian Ya-Wen Cheng, Chia-Ling Tu, Hsiu-Chun Chien, Hui-Chen Hsu, Shu-Mei Hong, Chiu-Mieh Huang, Jong-Long Guo

**Affiliations:** 1 Department of Health Promotion and Health Education, College of Education, National Taiwan Normal University, Taipei 106308, Taiwan; 2 Institute of Health Behaviors and Community Sciences, College of Public Health, National Taiwan University, Taipei 106319, Taiwan; 3 Institute of Clinical Nursing, College of Nursing, National Yang Ming Chiao Tung University, Taipei 112304, Taiwan

**Keywords:** augmented reality, digital health, aromatherapy, dementia, behavioral and psychological symptoms of dementia, non-pharmacological intervention

## Abstract

**Highlights:**

**What are the main findings?**

- Greater Reduction in BPSD and Caregiver Distress Compared with Routine Care: Participants receiving the 12-session AR-based aromatherapy education program showed significantly greater reductions in the frequency and severity of BPSD, as well as caregiver distress, than those receiving routine care.
- Improvements in Physical and Psychosocial Outcomes: Compared with routine care, the intervention was associated with significant improvements in pain, insomnia severity, subjective well-being, and life satisfaction.
- Potential of AR-Integrated Aromatherapy as a Non-Pharmacological Approach: The findings suggest that the AR-based aromatherapy education program may offer a promising non-pharmacological approach for supporting the care of older adults with early-stage dementia.

**What are the implications of the main findings?**

- Clinical Relevance of AR-Integrated Aromatherapy in Dementia Care: These findings suggest that AR-based aromatherapy education program may be clinically relevant as a technology-enhanced, non-pharmacological approach for reducing neuropsychiatric symptoms and improving well-being among older adults with early-stage dementia.
- Potential Relevance for Institutional Dementia Care: The intervention may have practical relevance for long-term care and related therapeutic settings, with the potential to reduce caregiver burden and support the delivery of dementia care.

**Abstract:**

Background: Behavioral and psychological symptoms of dementia (BPSD) substantially impair quality of life in individuals with dementia and increase caregiver burden. Conventional non-pharmacological interventions may be limited in terms of engagement and individualized support. This study evaluated the effects of an augmented reality (AR)-integrated aromatherapy intervention on BPSD and psychosocial well-being among older adults with early-stage dementia. Methods: A randomized controlled trial was conducted with 160 participants in Taiwan, including an experimental group (*n* = 80) and a comparison group (*n* = 80). The experimental group received a 6-week intervention comprising 12 AR-based aromatherapy sessions delivered twice weekly, whereas the comparison group received routine care. Generalized estimating equations (GEEs) were used to examine group-by-time interaction effects while controlling for baseline gender. Results: Significant group-by-time interaction effects were observed across all outcome measures (all *p* < 0.001). Compared with the comparison group, the experimental group showed greater reductions in NPI-Q symptom frequency (β = −0.775), symptom severity (β = −2.575), and caregiver distress (β = −4.800). In addition, the intervention group demonstrated significant improvements in pain (β = −2.625), insomnia severity (β = −4.425), psychological well-being (β = 7.675), and life satisfaction (β = 6.913). Baseline gender differences did not significantly affect intervention outcomes. Conclusions: The AR-integrated aromatherapy intervention appears to be an effective non-pharmacological approach for reducing BPSD and improving physical and psychosocial well-being among older adults with early-stage dementia. This technology-enhanced intervention may offer a promising care model for dementia management in geriatric care settings.

## 1. Introduction

Dementia represents a rapidly escalating global health challenge, imposing a profound burden not only on affected individuals and their caregivers but also on societal structures and healthcare systems [1]. As populations age worldwide, the complexity of managing this neurodegenerative condition has become a public health priority. In Taiwan, the prevalence among adults aged 65 and older reached 7.99% in 2025. This trend is projected to rise from 350,000 in 2024 to double to 680,000 by 2041, signaling an urgent and growing need for scalable, effective interventions to support this population [2].

In addition to cognitive deficits, Behavioral and Psychological Symptoms of Dementia (BPSD), including agitation, aggression, depression, anxiety, and sleep disturbances, affect 66.01% of dementia patients, significantly increasing both caregiver stress and healthcare expenditures [2]. BPSD does not merely increase caregiver burden; it is also intricately linked to physical pain, insomnia, and declining subjective well-being and life satisfaction in a vicious cycle [1,2]. These neuropsychiatric manifestations are often more debilitating than memory loss alone, as they significantly exacerbate caregiver stress, diminish the patient’s quality of life, and drastically increase healthcare expenditures. It is a critical predictor of emergency care and hospitalization, and using a validated metric like the Neuropsychiatric Inventory Questionnaire (NPI-Q) is substantial. This tool allows for the systematic quantification of neuropsychiatric severity alongside the measurement of caregiver distress, facilitating more targeted interventions [1].

However, the rapid evolution of digital health technology has introduced transformative potential to augment these traditional care models. Rapid advancements in digital health have catalyzed the development of specialized technological solutions, such as augmented reality (AR), virtual reality (VR), and social robotics, aimed at addressing the multifaceted gaps in dementia care.

Among emerging solutions, AR has shown promise as a versatile modality. By superimposing digital overlays onto the user’s physical environment, it can provide intuitive cognitive prompts and behavioral support while preserving sensory engagement with the immediate surroundings [3]. Within this digital landscape, AR has distinguished itself as an exceptionally versatile modality; by superimposing digital overlays onto the user’s physical surroundings, it delivers intuitive cognitive prompts and behavioral scaffolding while maintaining the user’s sensory connection to their immediate environment. This “anchored” interaction reduces the risks of sensory disconnect and cyber-sickness often associated with fully immersive VR, rendering AR a relatively safer and accessible medium for health education and cognitive stimulation in older populations [4].

Parallel to technological advancements, non-pharmacological interventions have gained significant attention due to the limited efficacy and potential side effects of psychotropic medications in older populations with dementia. Aromatherapy, in particular, has been empirically demonstrated to modulate the autonomic nervous system and alleviate BPSD symptoms through the inhalation of essential oils [5]. Specifically, aromatherapy has been demonstrated to effectively mitigate the behavioral and psychological symptoms of dementia. While numerous studies have integrated AR into cognitive training and health care, and aromatherapy has shown promising results in symptom management, there remains a significant gap in the literature regarding the integration between these two modalities [6,7].

To bridge the gap between technology and healthcare, this study proposes an integrated intervention that combines digital visual guidance with sensory-based therapeutic support. By leveraging AR technology to provide immersive, step-by-step interactive guidance, the intervention facilitates the delivery of aromatherapy, an olfactory-based approach associated with emotional regulation. This dual-sensory intervention was designed to address the multifaceted needs of dementia care, including patient safety, caregiver psychological well-being, and a supportive home environment.

Accordingly, this randomized controlled trial was conducted to evaluate the effects of an AR-based aromatherapy education program on BPSD and psychosocial well-being among older adults with early-stage dementia. In addition, the study explored the potential applicability of this technology-enhanced intervention in dementia care. By integrating AR-based guidance with aromatherapy, this study aimed to examine whether such an approach may provide a feasible non-pharmacological option in geriatric care.

## 2. Materials and Method

### 2.1. Study Design

This study used a two-arm randomized controlled trial with a parallel-group design to evaluate the effects of an AR-based aromatherapy education program on BPSD. The intervention targeted older adults with dementia and their primary caregivers. Outcomes were measured at baseline (T1) and at 6 weeks after the intervention (T2).

A total of 160 participants were enrolled and randomly assigned to the experimental group (*n* = 80) or the comparison group (*n* = 80). Simple randomization using a random number table was applied to ensure equal allocation probability and minimize selection bias. Due to the practical constraints of the community-based setting, the primary researcher was responsible for both enrolling participants and assigning them to their respective intervention groups; consequently, the random allocation sequence was accessible to the individual managing the enrollment process. To mitigate potential bias arising from this open-allocation process, the study implemented a single-blind approach where possible. Due to the nature of the intervention (interactive AR and fragrant essential oils), it was not possible to blind the participants or the primary interventionist to the group assignments after the allocation. However, to maintain the integrity of the data, the outcome assessors and data analysts remained blinded to the group assignments throughout the study duration. This measure was taken to ensure that the evaluation of BPSD symptoms and psychosocial outcomes remained objective and independent of the intervention delivery. The experimental group received the AR-based aromatherapy education program, whereas the comparison group received routine care. Because of the nature of the intervention, participant blinding was not possible; however, research assistants conducting follow-up assessments were blinded to group allocation to reduce detection bias. Data collection for this study started on 23 May 2025, with the primary intervention and follow-up assessments reaching completion on 30 October 2025.

A prior power analysis using SPSS version 27.0 indicated that a minimum of 64 participants per group was required to detect a medium effect size (Cohen’s d = 0.50) with 80% power at a significance level of 0.05 [8,9]. To consider for potential 20% attrition and mortality in this older population, 160 participants were enrolled and randomized (80 per group). This sample size was considered adequate to preserve statistical power throughout the trial. In addition, the use of Generalized Estimating Equations (GEE) allowed for longitudinal analysis using all available observations.

### 2.2. Participants

The study cohort consisted of adults aged 65 years and older who were recruited from community centers, long-term care facilities, and adult day care centers across Taiwan. Recruitment was conducted through a multi-site approach. The research team first contacted these institutions to explain the study objectives and eligibility criteria. After potential participants were identified with institutional assistance, trained research personnel conducted on-site screenings to confirm eligibility according to predefined criteria.

Inclusion criteria included an AD-8 score of 2 or higher, indicating cognitive concern, which is widely recognized and utilized in Taiwan’s public health system for large-scale community screening to identify older adults requiring early supportive care [10,11]. Participants were also required to demonstrate the ability to acquire basic technology-related skills and to have adequate visual, auditory, and olfactory function to participate fully in the intervention. In addition, at least a junior high school level of education was required to ensure comprehension of the assessment instruments. Exclusion criteria included diagnosed psychiatric disorders, unstable chronic physical conditions, and moderate to severe dementia. Before formal enrollment, research staff provided all potential participants and their primary caregivers with an explanation of the study purpose, procedures, and potential risks and benefits. Written informed consent was obtained from all participants prior to baseline data collection.

### 2.3. Study Procedure

**Figure 1:**
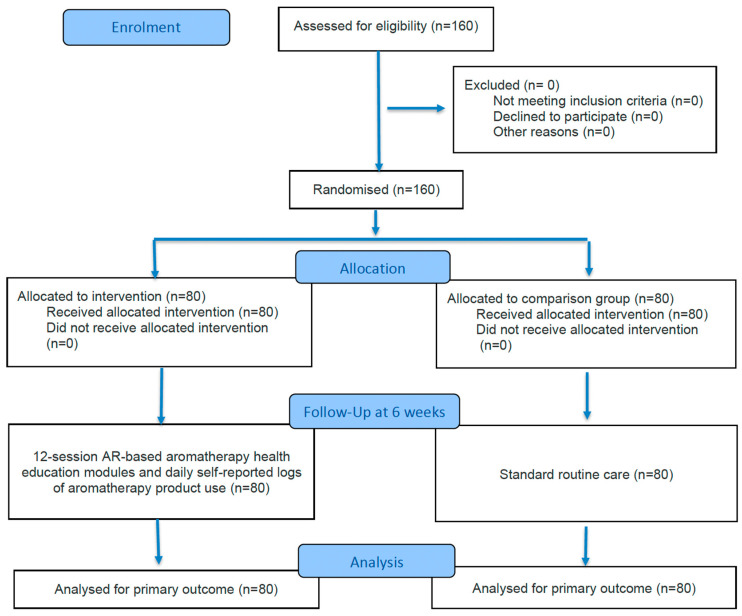
CONSORT 2025 Flowchart of participant recruitment and intervention.

The flowchart of the trial is presented in Figure 1, and the study is reported in accordance with the CONSORT 2025 statement (see Supplementary Materials) [12]. Before the trial began, participants and their primary caregivers attended an orientation session conducted by trained research staff, during which the study aims, procedures, and intervention process were explained. Written informed consent was then obtained from all participants and their primary caregivers prior to enrollment. Eligible participants were subsequently randomly assigned to either the experimental group or the comparison group using a random number table, ensuring an equal probability of assignment and reducing the risk of selection bias.

The experimental group received an AR-based aromatherapy education program targeting 12 BPSD-related symptoms. Rather than focusing solely on the individual as the unit of analysis, this study adopted a symptom-level approach to reflect the clinical presentation of dementia, in which multiple symptoms commonly occur concurrently. After baseline assessment, participants in the experimental group completed a structured 12-session program over 6 weeks, with two sessions per week. Each session focused on a specific BPSD-related symptom and used a tablet-based AR interface to deliver interactive instruction on essential oil characteristics and application methods.

During this phase, a sequential symptom-matched intervention was implemented. For each primary symptom identified at baseline assessment (T1), a corresponding aromatherapy product was provided for 2 weeks of home use. Because most participants exhibited multiple concurrent symptoms, the full sequence of interventions was typically completed within the 6-week study period. Participants were also asked to maintain a daily log of aromatherapy use and symptom changes. The comparison group received standard health education materials routinely provided by their community centers or long-term care facilities.

To address potential safety concerns, we implemented a multi-layered monitoring approach. First, the aromatherapy intervention was non-invasive, which carries minimal risk of adverse reactions. Second, during the orientation and the initial week, participants were provided with a trial application of the products; no signs of skin irritation or respiratory discomfort were observed after 1–2 days of use. Third, trained facilitators monitored participants during each session, and caregivers used daily logs to record any physiological changes or discomfort at home. We confirm that throughout the six-week intervention, no participants reported or exhibited any allergic reactions, skin irritation, or respiratory symptoms related to the aromatherapy ingredients.

### 2.4. Intervention Program

The activities included in the AR-based aromatherapy education program were developed through collaboration among a multidisciplinary team consisting of dementia care specialists, professional aromatherapists, and engineers. The intervention was designed to support participant autonomy through structured cognitive guidance and sensory-based symptom management. The 12 sessions were developed in accordance with evidence-based dementia care principles and aligned with the 12 symptom domains of the Neuropsychiatric Inventory Questionnaire (NPI-Q) [1]. As shown in Table 1, the program focused on key objectives, including improving symptom awareness, enhancing aromatherapy-related skills, and supporting emotional self-regulation.

Each educational session was developed based on 12 common BPSD symptoms observed in individuals with early-stage dementia, as identified by the clinical professionals on the research team. The sessions were organized into narrative-based modules that reflected everyday stressors, such as sundowning-related agitation and nighttime restlessness, and presented aromatherapy as a potential supportive strategy. Each session included clearly defined learning objectives and formed part of a sequential learning pathway designed to enhance participant engagement and retention through scenario-based storytelling (Table 1).

**Figure 2:**
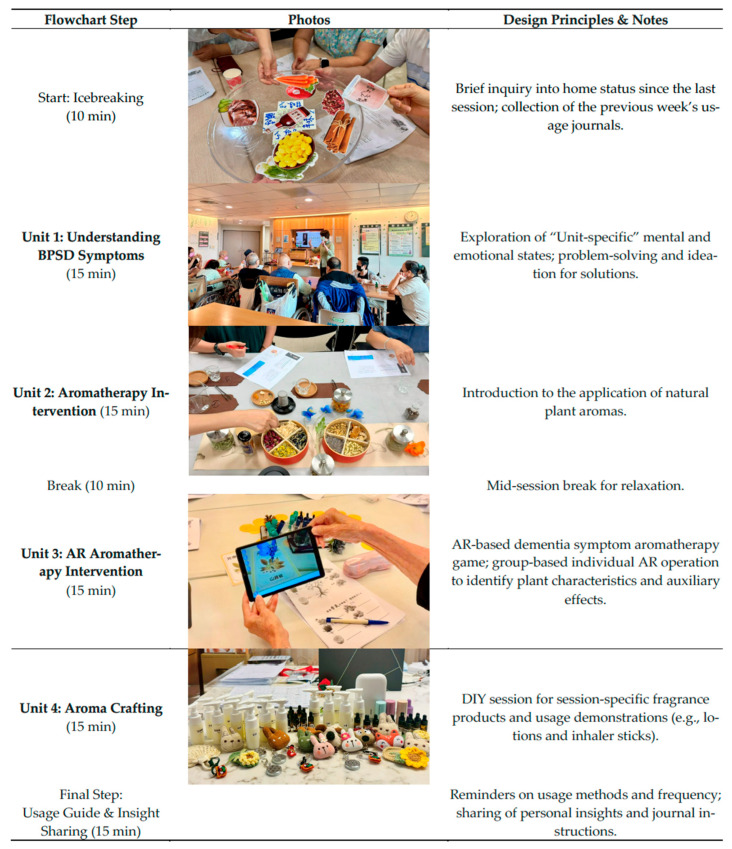
Sample session content and activities.

As shown in Figure 2, each session was structured to promote consistent learning and routine formation. The session began with a 10 min icebreaking and review phase, during which facilitators asked about participants’ emotional and physical conditions at home and collected usage logs to monitor adherence. This was followed by a 15 min symptom exploration segment, in which participants engaged in guided discussions to recognize how specific neuropsychiatric symptoms affected their daily lives. A 15 min theoretical component was then provided to introduce the proposed physiological mechanisms of aromatherapy, including how selected essential oil constituents may influence the nervous system in ways that could help relieve BPSD-related symptoms, while also offering practical guidance on the use of natural botanical essences.

After a scheduled 10 min break to reduce cognitive fatigue, participants proceeded to the interactive AR application phase. Developed by a multidisciplinary team of AR software engineers, professional aromatherapists, and dementia care specialists, the system was delivered via handheld tablets to ensure portability and ease of use in multi-site community settings. Utilizing a QR code-based trigger system on cards corresponding to the 12 symptom domains, the program created an interactive learning environment in which digital overlays were superimposed onto the user’s physical surroundings. The central component of this phase consisted of a 15 min AR-enhanced activity in which participants engaged in a gamified, spatially oriented task to identify plant characteristics. The AR interface supported this process through intuitive hotspots that enabled interaction with digital representations of botanical elements and their associated properties. This hands-on activity encouraged active participation and helped reinforce the association between visual cues and aromatic applications. To support motivation and task accuracy, gamified quizzes were embedded within the module to provide immediate audio and visual feedback. This real-time feedback was intended to reinforce correct symptom–oil associations and provide gentle instructional prompts when errors occurred, thereby supporting participants’ learning and self-management skills.

The practical component then included a 15 min hands-on aromatherapy preparation session, during which participants created symptom-specific products following step-by-step demonstrations. Each session concluded with a 15 min reflection and sharing period, in which trained facilitators used concept mapping techniques to help participants summarize key learning points and relate the activities to their own experiences.

Facilitators monitored learning progress throughout the sessions and provided technical and emotional support, particularly for participants who were less familiar with digital interfaces. The program underwent iterative refinement to enhance content validity, with all modules reviewed by two dementia care specialists and two senior health educators. Feedback from a prior pilot feasibility study was also incorporated to improve interface usability and comfort for older adults with cognitive concerns.

### 2.5. Measures

The primary and secondary outcomes were assessed at baseline (T1) and at 6-week post-intervention (T2). At baseline, sociodemographic characteristics of participants with dementia were collected, including age, gender, marital status, educational level, occupation, self-rated health status, history of seeking medical consultation for dementia-related symptoms, and prior cognitive function assessment. Outcome measures included primary outcomes, such as the 12 BPSD symptoms, their severity, and caregiver distress, as well as secondary outcomes, including pain intensity, insomnia severity, subjective well-being (WHO-5), and overall life satisfaction.

#### 2.5.1. Neuropsychiatric Inventory Questionnaire (NPI-Q)

The primary outcomes were assessed using the Neuropsychiatric Inventory Questionnaire (NPI-Q) [1], a widely used instrument for evaluating 12 neuropsychiatric symptoms commonly observed in dementia. These domains include delusions, hallucinations, agitation/aggression, depression/dysphoria, anxiety, elation/euphoria, apathy/indifference, disinhibition, irritability/lability, aberrant motor behavior, nighttime behavioral disturbances, and appetite/eating changes. Caregivers help identified whether each symptom was present. For symptoms that were present, severity was rated on a 3-point scale (1 = mild, 2 = moderate, 3 = severe). Caregivers also rated the level of distress associated with each symptom on a 6-point Likert scale ranging from 0 (no distress) to 5 (extreme distress).

#### 2.5.2. Pain

Pain intensity was assessed using the Abbey Pain Scale [13], an observational instrument validated for use in individuals with dementia who may have difficulty communicating pain verbally. The scale comprises six items assessing behavioral and physiological indicators, including vocalization, facial expression, body language, behavioral changes, physiological changes, and physical changes. Each item is scored from 0 to 3, yielding a total score ranging from 0 to 18. Total scores are categorized as no pain (0–2), mild pain (3–7), moderate pain (8–13), or severe pain (14 or above).

#### 2.5.3. Insomnia

Insomnia severity was assessed using the Athens Insomnia Scale (AIS) [14]. The AIS is an 8-item self-report instrument that assesses sleep induction, awakenings during the night, final awakening, total sleep duration, overall sleep quality, well-being during the day, functioning during the day, and daytime sleepiness. Each item is rated on a 4-point scale (0–3), with higher total scores indicating more severe insomnia symptoms.

#### 2.5.4. Subjective Well-Being (WHO-5)

Subjective well-being was assessed using the WHO-5 Well-Being Index [15]. This 5-item instrument evaluates positive well-being over the previous 2 weeks. Respondents rated items such as “I have felt cheerful and in good spirits” on a 6-point scale ranging from 0 (at no time) to 5 (all of the time). The WHO-5 has been widely used in clinical research to assess changes in well-being.

#### 2.5.5. Life Satisfaction

Overall life satisfaction was assessed with the Satisfaction With Life Scale (SWLS) [16]. This 5-item instrument measures an individual’s global cognitive evaluation of overall life satisfaction. Participants rated each item on a 7-point Likert scale ranging from 1 (strongly disagree) to 7 (strongly agree). Higher scores indicate greater life satisfaction.

### 2.6. Statistical Analysis

All statistical analyses were performed using IBM SPSS Statistics version 27.0. Descriptive statistics were used to summarize baseline demographic characteristics and clinical variables. Independent *t*-tests and Chi-square tests were conducted to assess baseline comparability between the experimental and comparison groups. Within-group changes from T1 to T2 were tested using paired *t*-tests, and effect sizes for all outcomes were calculated. Longitudinal effects of the intervention at T1 and T2 were examined using Generalized Estimating Equations (GEE), with a group-by-time interaction term included to test whether changes in outcomes differed between groups over time. Regression coefficients (β), standard errors (SE), and Wald chi-square statistics were reported for each outcome. A two-tailed *p*-value < 0.05 was considered statistically significant.

### 2.7. Ethical Considerations

The study was conducted in accordance with the Declaration of Helsinki and approved by the Institutional Review Board of National Taiwan Normal University (Approval No. 202212HM033). In accordance with international standards for reporting clinical trials, the study was prospectively registered at ClinicalTrials.gov (Registry Name: Augmented Reality-Based Aromatherapy Education for Older Adults With Early-Stage Dementia in Taiwan; Identifying Number: NCT07431164; URL: https://clinicaltrials.gov/study/NCT07431164, accessed on 22 March 2026). The registration was last updated on 9 March 2026, ensuring transparency and public access to the trial’s protocol and outcomes.

## 3. Results

### 3.1. Participant Characteristics

A total of 160 participants were included in the final analysis, with 80 assigned to the experimental group and 80 to the comparison group. All participants completed the full intervention and assessments, resulting in a 0% dropout rate, which resulted from the stable day care center environment and the short six-week intervention period, during which no participants relocated or passed away. Table 2 presents the baseline demographic and clinical characteristics of the study population. No significant between-group differences were observed in age group (χ^2^ = 2.713, *p* = 0.258), marital status (χ^2^ = 7.553, *p* = 0.056), educational level (χ^2^ = 1.863, *p* = 0.761), occupation (χ^2^ = 0.340, *p* = 0.560), self-rated health status (χ^2^ = 1.983, *p* = 0.576), previous medical consultation for dementia-related symptoms (χ^2^ = 0.400, *p* = 0.527), or prior cognitive assessment (χ^2^ = 0.101, *p* = 0.751). A significant between-group difference was observed for gender (χ^2^ = 5.942, *p* = 0.015); therefore, gender was included as a covariate in the subsequent GEE analyses.

### 3.2. Within-Group Comparison

Paired *t*-tests within groups were conducted to evaluate the changes within each group from baseline (T1) to post-intervention (T2). As presented in Table 3, participants in the experimental group showed significant improvements across all outcome variables. Regarding BPSD, participants exhibited a marked decrease in symptoms (*t* = 3.47, *p* < 0.001), severity (*t* = 8.50, *p* < 0.001), and caregiver distress (*t* = 10.17, *p* < 0.001). Physical pain levels significantly decreased (*t* = 5.71, *p* < 0.001), and insomnia as well (*t* = 7.85, *p* <.001). Furthermore, participants reported significant gains in well-being (*t* = 9.29, *p* < 0.001) and life satisfaction (*t* = 7.74, *p* < 0.001). The positive coefficients for NPI-Q symptoms, severity, caregiver distress, pain, and insomnia, and negative coefficients for well-being and life satisfaction, indeed reflect a deterioration in the comparison group after 6 weeks.

**Figure 3:**
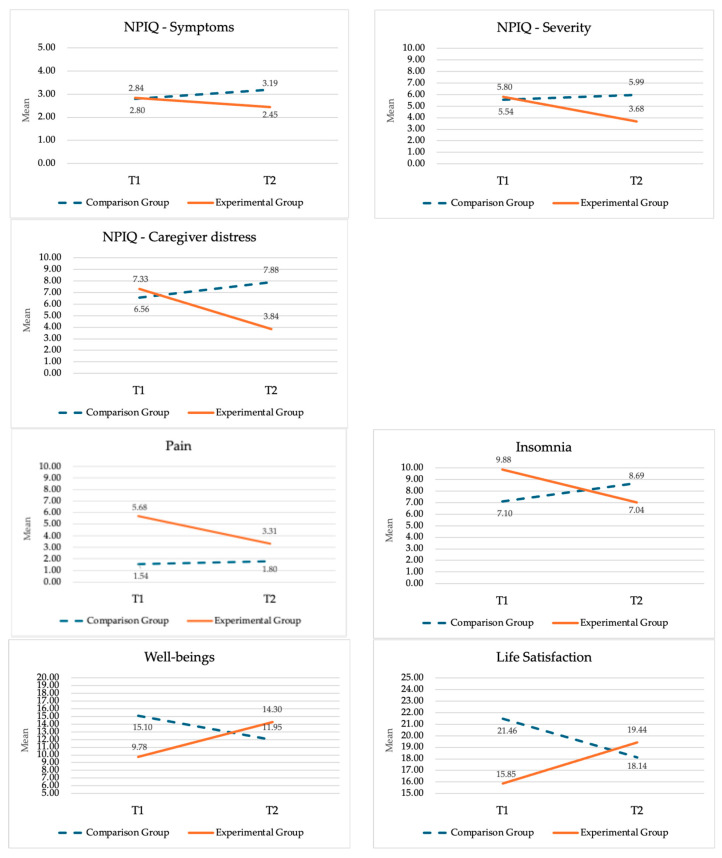
Changes in outcome variables at T1 and T2.

Similarly, Figure 3 presents the changes in the mean scores of outcome variables at T1 and T2. Table 4 presents the Cohen’s d effect sizes and 95% confidence intervals (CI) for both groups, providing further insight into the clinical magnitude of the observed changes from T1 to T2.

### 3.3. Intervention Effects

The GEE results (Table 5) showed significant group-by-time interaction effects for all primary and secondary outcomes after adjustment for baseline gender. For neuropsychiatric symptoms measured using the NPI-Q, the experimental group showed significantly greater reductions in symptom frequency (β = −0.775, *p* < 0.001), symptom severity (β = −2.575, *p* < 0.001), and caregiver distress (β = −4.800, *p* < 0.001) than the comparison group at the post-intervention assessment (T2).

Similar patterns were observed for the physiological and psychosocial outcomes. Compared with the comparison group, the experimental group showed greater reductions in pain intensity (β = −2.625, *p* < 0.001) and insomnia severity (β = −4.425, *p* < 0.001). In addition, the intervention was associated with significant improvements in psychological well-being (β = 7.675, *p* < 0.001) and life satisfaction (β = 6.913, *p* < 0.001), as reflected by the positive interaction coefficients. Although gender was included as a covariate, it was not significantly associated with outcome variables (*p* > 0.05), suggesting that the effects of the AR-based aromatherapy education program were generally consistent across gender.

## 4. Discussion

The study evaluated the effects of an augmented reality (AR)-integrated aromatherapy education program on behavioral and psychological symptoms of dementia (BPSD) and psychosocial well-being among Taiwanese older adults with early-stage dementia. The main findings from the GEE analysis showed that the 12-session intervention was associated with significantly greater improvements than routine care across all primary and secondary outcomes. Specifically, participants in the experimental group showed greater reductions in the frequency and severity of neuropsychiatric symptoms, along with lower levels of caregiver distress. The intervention was also associated with improvements in pain, insomnia severity, subjective well-being, and life satisfaction. The findings suggest that integrating AR-based guidance with aromatherapy may offer a useful non-pharmacological approach for addressing multiple care needs in older adults with early-stage dementia.

The findings indicate that the experimental group receiving the AR aromatherapy program demonstrated significantly better improvements compared to the comparison group receiving routine care. This aligns with existing literature, suggesting that technology-enhanced interventions can effectively counteract the natural functional decline common in this population. Notably, the comparison group exhibited deterioration in NPI-Q and caregiver distress scores after six weeks. This reflects the natural progression of neuropsychiatric symptoms and functional decline in older adults with potential dementia risk when specific support is absent. The stabilization and improvement seen in the experimental group underscore the intervention’s clinical value in delaying such deterioration [6,7].

The intervention was associated with reductions in BPSD frequency and severity, along with improvements in pain and insomnia severity. With regard to the role of AR, these findings are consistent with previous studies suggesting that AR-based cognitive support can provide structured and immersive cues that help maintain attention and promote task engagement in dementia care [4,17]. Recent systematic reviews have further indicated that technology such as AR, is feasible for use in cognitive rehabilitation for individuals with mild cognitive impairment and dementia, with potential benefits for memory, attention, executive function, and daily task performance [3,18].

With regard to aromatherapy, our findings on pain and sleep outcomes are broadly consistent with previous studies [5,19]. A 2024 meta-analysis of 15 trials involving 821 participants reported significant improvements in behavioral and psychological symptoms following 3 to 4 weeks of aromatherapy intervention [6]. In addition, recent evidence suggests that inhaled aromatherapy may improve cognitive performance, as reflected in Mini-Mental State Examination (MMSE) and Montreal Cognitive Assessment (MoCA) scores among individuals with cognitive impairment [7]. Although cognition was not measured in the present study, these findings may provide indirect support for the potential role of aromatherapy in dementia care.

Although cognitive function was not measured in the present study, existing research in dementia mice models has suggested that some of the essential oils used in this program may have potential neuroprotective effects, such as reducing amyloid-beta levels and tau phosphorylation. These findings provide an important biological context for the potential of aromatherapy in dementia care [20]. Furthermore, BPSD can be viewed as a component of geriatric syndromes, which often require multicomponent, non-pharmacological approaches to manage their complexity and support the independence of older adults [21]. By situating our findings within this broader clinical framework, we can better understand the role of AR-integrated aromatherapy as a supportive strategy for symptom alleviation rather than cognitive restoration.

In addition to improvements in symptom-related outcomes, the experimental group demonstrated significantly higher levels of well-being and life satisfaction. This finding suggests that aromatherapy may have broader psychosocial benefits beyond symptom relief, possibly through mechanisms related to relaxation and positive affect [7,22,23,24].

Meta-analyses have suggested that aromatherapy, particularly lavender oil, may reduce agitation and aggression in individuals with cognitive impairment, with short-term interventions (≤4 weeks) appearing to yield greater effects than longer treatment durations [24]. In a recent meta-analysis, 4 of 15 trials also reported improvements in depressive mood following aromatherapy intervention [7]. In addition, essential oil therapy combined with standard care has been associated with reductions in both BPSD and caregiver distress, without reported adverse effects. A randomized controlled trial further found that environmental diffusion of essential oils combined with psychotropic drug therapy was associated with significantly lower caregiver distress (*p* < 0.01) than standard pharmacological treatment alone [6]. The study also found a significant reduction in caregiver distress. This finding suggests that the potential benefits of AR-based aromatherapy may extend beyond the individual with dementia to the broader caregiving context. Reduced caregiver distress may also have practical relevance for dementia care by helping to ease the burden on both family and professional caregivers [6,25].

The reduction in both BPSD and caregiver distress observed in the present study is also consistent with the broader literature on multicomponent interventions. Interventions that combine education, skills training, and psychotherapeutic support have been reported to show favorable effects on both symptom outcomes and caregiver distress [26,27]. A 2025 network meta-analysis found that multicomponent interventions had the highest ranking for reducing behavioral and psychological symptoms of dementia and improving caregiver reactions to these symptoms [27]. This suggests that technology-enhanced aromatherapy may have benefits beyond the individual patient, with the potential to improve quality of life for both people with dementia and their caregivers [6,25].

Several limitations of this study should be acknowledged. First, because the program was designed as a multicomponent AR-supported aromatherapy education intervention, the individual contributions of the AR technology and the hands-on aromatherapy sessions cannot be easily isolated using the current study design, as they are intrinsically linked to each other [28]. This integration was intentional, reflecting a ‘human–technology collaboration’ model where AR serves as a motivational and instructional scaffold to facilitate the delivery of sensory therapy. To validate the effectiveness of these two approaches separately, future studies should consider using factorial designs or controlled trials with an active control arm and sufficient statistical power. Second, as a randomized controlled trial conducted in Taiwan, the relatively small sample size may limit the generalizability of the findings to other settings and populations. Third, although the 6-week intervention allowed for the assessment of short-term changes between baseline and post-intervention, the durability of the observed effects remains unclear. Previous reviews of immersive technology interventions for dementia have noted that small sample sizes and short follow-up periods remain important challenges in evaluating long-term effectiveness. In addition, the baseline gender imbalance was a chance result of randomization; while adjusted statistically, future studies should employ stratified randomization. Finally, the outcome measures were based primarily on self-reported and caregiver-reported assessments, which may be subject to reporting bias. Due to the nature of the active interventions, it was practically impossible to blind the participants and the primary interventionist to the group allocation. This lack of double-blinding could introduce potential performance and expectancy biases.

Several limitations of this study should be acknowledged. First, although the 6-week intervention allowed for the assessment of short-term changes between baseline and post-intervention, the durability of the observed effects remains unclear. Previous reviews of immersive technology interventions for dementia have noted that small sample sizes and short follow-up periods remain critical challenges in evaluating long-term effectiveness. Second, the outcome measures were based primarily on self-reported and caregiver-reported assessments, which may be subject to reporting bias. Future studies should therefore include larger samples, longer follow-up periods, and more objective outcome measures in addition to subjective assessments. More consistent reporting of key participant characteristics and adverse events would also strengthen the evidence base.

## 5. Conclusions

In conclusion, this study suggests that an AR-integrated aromatherapy intervention may improve well-being among older adults with early-stage dementia. The findings indicate that combining digital technology with sensory-based approaches may represent a promising non-pharmacological strategy for dementia care.

## Figures and Tables

**Table 1:** AR-based Aromatherapy Education module.

Session	NPI-Q Symptom	Aromatherapy Activity	Learning Objectives
1	Delusion	Aroma necklace crafting	Facilitating cognitive clarity and orientation Divert attention
2	Hallucination	Aroma Massage oil blending	Enhancing reality orientation Alleviating agitation
3	Agitation or aggression	Aroma lotion crafting	Mitigating irritability and anxiety Enhance comfort Ameliorating Sundown Syndrome-related agitation
4	Depression or dysphoria	Eco-printing	Fostering social interaction and engagement Boosting self-efficacy and confidence Emotional regulation
5	Anxiety	Aroma ointment crafting	Stress reduction Relieve tension and anxiety
6	Elation or euphoria	Aroma Sachet crafting	Consolidating attentional focus Maintaining visuospatial and hand-eye coordination Emotional regulation
7	Apathy or indifference	Creative floral art	Providing psychosocial and emotional support Strengthening life perception and sensory awareness Enhancing self-esteem and subjective life satisfaction
8	Disinhibition	Herb picking/Tea bagging	Mitigating agitation and irritability Emotional stabilization Divert attention
9	Irritability or lability	Aroma nasal inhaler crafting	Emotional stabilization Psychosomatic relaxation Divert attention
10	Motor disturbance	Aroma bath salt crafting	Reducing agitation and restlessness Mitigating motor disturbance Enhancing a sense of comfort and well-being
11	Nighttime behaviors	Lavender eye pillow making	Alleviating agitated behaviors Improving sleep disturbances and reducing nocturnal behaviors
12	Appetite and eating	Aroma diffuser spray crafting	Stabilizing and redirecting negative affect Adjusting diet and appetite

**Table 2:** Demographic results of participants (*n* = 160).

Variables	Comparison Group (*n* = 80, 50%)		Experimental Group (*n* = 80, 50%)		χ^2^/*t*	*p* Value
Age (years)					2.713	0.258
65–74	19	23.8	16	20.0		
75–84	37	46.3	30	37.5		
85+	24	30.0	34	42.5		
Gender, n (%)					5.942	0.015
Male	25	31.3	12	15.0		
Female	55	68.7	68	85.0		
Marital status, n (%)					7.553	0.056
Unmarried	3	3.8	1	1.3		
Married	27	33.8	23	28.7		
Divorced or separated	9	11.2	2	2.5		
Widowed	41	51.2	54	67.5		
Educational level, n (%)					1.863	0.761
Elementary school	46	57.5	41	51.2		
Middle school	16	20.0	19	23.8		
High school	13	16.3	11	13.8		
University and above	5	6.3	9	11.2		
Occupation, n (%)					0.340	0.560
Yes	2	2.5	1	1.2		
No	78	97.5	79	98.8		
Self-rated health status, n (%)					1.983	0.576
Very poor	7	8.7	8	10.0		
Poor	47	58.8	50	62.5		
Good	22	27.5	21	26.3		
Very good	4	5.0	1	1.3		
Sought medical consultation for dementia symptoms, n (%)					0.400	0.527
Yes	37	46.2	41	51.2		
No	43	53.8	39	48.8		
Undergone a cognitive function assessment, n (%)					0.101	0.751
Yes	43	53.8	45	56.2		
No	37	46.2	35	43.8		

**Table 3:** Paired *t*-tests within groups.

	Comparison Group						Experimental Group					
	T1		T2				T1		T2			
Variables	Mean	SD	Mean	SD	*t*	*p*	Mean	SD	Mean	SD	*t*	*p*
NPIQ—Symptoms	2.80	2.29	3.19	2.42	2.11	0.038	2.84	1.67	2.45	1.36	3.47	<0.001
NPIQ—Severity	5.54	4.51	5.99	4.48	−1.61	0.111	5.80	3.83	3.68	3.11	8.50	<0.001
NPIQ—Caregiver distress	6.56	6.05	7.88	6.90	3.71	<0.001	7.33	5.86	3.84	4.70	10.17	<0.001
Pain	1.54	2.21	1.80	2.30	−1.31	0.194	5.68	5.24	3.31	3.40	5.71	<0.001
Insomnia	7.10	5.48	8.69	5.31	3.90	<0.001	9.88	4.68	7.04	4.18	7.85	<0.001
Well-beings	15.10	6.59	11.95	6.14	5.39	<0.001	9.78	6.40	14.30	5.18	9.29	<0.001
Life Satisfaction	21.46	8.02	18.14	7.68	5.20	<0.001	15.85	6.35	19.44	5.21	7.74	<0.001

**Table 4:** Effect Size.

	Comparison Group	Experimental Group
Variables	d (95% CI)	d (95% CI)
NPIQ—Symptoms	0.24 (0.01–0.46) *	0.39 (0.16–0.61) ***
NPIQ—Severity	−0.18 (−0.40–0.04)	0.95 (0.68–1.21) ***
NPIQ—Caregiver distress	0.42 (0.19–0.64) ***	1.14 (0.85–1.42) ***
Pain	−0.15 (−0.37–0.07)	0.64 (0.40–0.88) ***
Insomnia	0.44 (0.21–0.66) ***	0.88 (0.62–1.13) ***
Well-beings	0.60 (0.36–0.84) ***	1.04 (0.76–1.31) ***
Life Satisfaction	0.58 (0.34–0.82) ***	0.87 (0.61–1.12) ***

Note: * *p* < 0.05; *** *p* < 0.001.

**Table 5:** GEE ^a^ results for interventions.

Variable	Coefficient (β)	SE	*p*-Value
NPIQ—Symptoms (0–12 points)			
Intercept	2.445	0.1362	<0.001
Gender	0.034	0.4557	0.941
Time (T2) ^b^	0.388	0.1111	<0.001
Group (Experimental group) ^c^	0.732	0.3313	0.027
Time (T2) × Group (Experimental group) ^d^	−0.775	0.2136	<0.001
NPIQ—Severity (0–36 points)			
Intercept	3.658	0.2873	<0.001
Gender	0.113	0.9243	0.903
Time (T2) ^b^	2.125	0.2484	<0.001
Group (Experimental group) ^c^	2.294	0.6908	<0.001
Time (T2) × Group (Experimental group) ^d^	−2.575	0.3726	<0.001
NPIQ—Caregiver distress (0–60 points)			
Intercept	3.854	0.4149	<0.001
Gender	−0.107	1.4195	0.940
Time (T2) ^b^	3.488	0.3410	<0.001
Group (Experimental group) ^c^	4.055	1.0818	<0.001
Time (T2) × Group (Experimental group) ^d^	−4.800	0.4898	<0.001
Pain (0–18 points)			
Intercept	3.412	0.3772	<0.001
Gender	−0.662	0.5475	0.226
Time (T2) ^b^	2.363	0.4113	<0.001
Group (Experimental group) ^c^	−1.405	0.4853	0.004
Time (T2) × Group (Experimental group) ^d^	−2.625	0.4569	<0.001
Insomnia (0–24 points)			
Intercept	7.187	0.4794	<0.001
Gender	−0.997	0.8798	0.257
Time (T2) ^b^	2.838	0.3590	<0.001
Group (Experimental group) ^c^	1.812	0.7693	0.019
Time (T2) × Group (Experimental group) ^d^	−4.425	0.5412	<0.001
Well-beings (0–25 points)			
Intercept	14.032	0.6002	<0.001
Gender	1.786	1.0373	0.085
Time (T2) ^b^	−4.525	0.4841	<0.001
Group (Experimental group) ^c^	−2.640	0.9220	0.004
Time (T2) × Group (Experimental group) ^d^	7.675	0.7564	<0.001
Life Satisfaction (5–35 points)			
Intercept	19.258	0.6154	<.001
Gender	1.197	1.0815	0.269
Time (T2) ^b^	−3.587	0.4609	<0.001
Group (Experimental group) ^c^	−1.494	1.0800	0.166
Time (T2) × Group (Experimental group) ^d^	6.913	0.7845	<0.001

Note: ^a^ GEE: generalized estimating equation; ^b^ Reference group (Time): Time (T1); ^c^ Reference group (Group): Comparison group; ^d^ Reference group (Time × Group): Time (T1) × Comparison group.

## Data Availability

The data presented in this study are available on request from the corresponding author. The data are not publicly available due to privacy or ethical restrictions.

## Supplementary Materials

The following supporting information can be downloaded at: https://www.mdpi.com/article/10.3390/healthcare14111482/s1, CONSORT 2025 checklist of information to include when reporting a randomised trial [12].
